# Is the brain really a small-world network?

**DOI:** 10.1007/s00429-015-1035-6

**Published:** 2015-04-18

**Authors:** Claus C. Hilgetag, Alexandros Goulas

**Affiliations:** Department of Computational Neuroscience, University Medical Center Hamburg-Eppendorf, Martinistr. 52, 20246 Hamburg, Germany; Department of Health Sciences, Boston University, 635 Commonwealth Ave., Boston, MA 02215 USA; Max Planck Research Group Neuroanatomy and Connectivity, Max Planck Institute for Human Cognitive and Brain Sciences, Stephanstraße 1A, 04103 Leipzig, Germany

**Keywords:** Brain connectivity, Connectome, Hierarchical modular networks, Large-world networks

## A matter of network topology

It is commonly assumed that the brain is a small-world network (e.g., Sporns and Honey 2006). Indeed, one of the present authors claimed as much 15 years ago (Hilgetag et al. 2000). The small-worldness is believed to be a crucial aspect of efficient brain organization that confers significant advantages in signal processing (e.g., Lago-Fernández et al. 2000). Correspondingly, the small-world organization is deemed essential for healthy brain function, as alterations of small-world features are observed in patient groups with Alzheimer’s disease (Stam et al. 2007), autism (Barttfeld et al. 2011) or schizophrenia spectrum diseases (Liu et al. 2008; Wang et al. 2012; Zalesky et al. 2011).

**Fig. 1:**
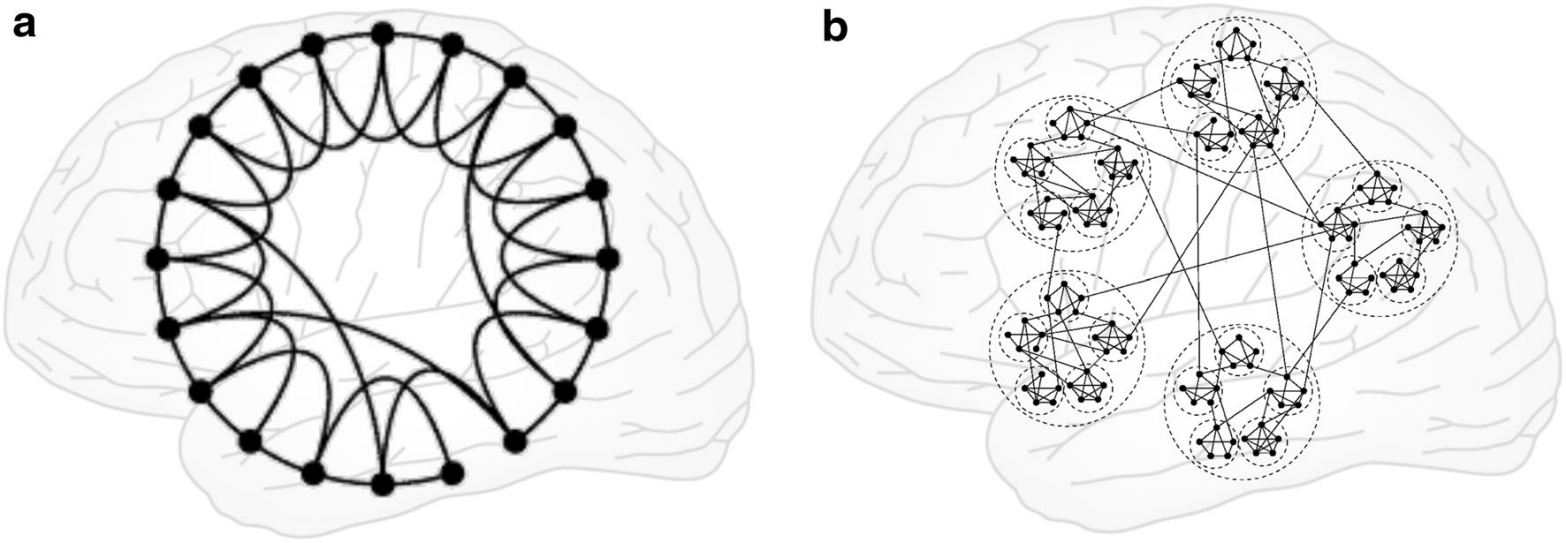
Classical small-world network (**a**) versus hierarchical modular network (**b**). Classical small-world networks can be derived by partial random rewiring of regular networks, which results in high clustering and relatively short path lengths. While hierarchical modular networks may also possess these features, they can also be large-world networks with a finite topological dimension. This aspect makes them an intriguing model for brain networks. Adapted from Watts and Strogatz (1998) and Kaiser et al. (2007)

While the colloquial idea of a small, interconnected world has a long tradition (e.g., Klemperer 1938), the present concept of small-world features of networks is frequently associated with the Milgram experiment (Milgram 1967) that demonstrated surprisingly short paths across social networks (‘six degrees of separation’). The concept was formalized by Watts and Strogatz (1998), who derived small-world networks from regular networks by including a small proportion of random network shortcuts. Such an organization results in short paths across the whole network—almost as small as in random networks—combined with local ‘cliquishness’ (or clustering) of neighboring nodes, due to dense local interconnections. These features can be mathematically summarized by the small-world coefficient (Humphries et al. 2006), which is defined as the clustering coefficient of a given network (normalized by the clustering coefficient of a same-size random network) divided by the network’s normalized average shortest pathlength. While any network that has a small-world coefficient larger than one is formally a small-world network, for many researchers, the term has become associated with the specific Watts and Strogatz model that is based on the partial random rewiring of a regular network (Fig. 1a). Indeed, the estimation of the rewiring probability has been used to directly associate real-world networks with the Watts and Strogatz model (Humphries and Gurney 2008). Incidentally, the small-world coefficient might not faithfully capture the small-world property as originally described by Watts and Strogatz (1998). Therefore, an alternative coefficient has been proposed that compares the clustering of the network to a lattice instead of a random network (Telesford et al. 2011).

A large number of empirical network data conform to the small-world features of short paths combined with high clustering, including many neural networks—but do these features capture the essence of the topological organization of brain networks? Real neural networks are very different from both regular and random networks; thus, a small-world organization, which by Watts and Strogatz’s (1998) model may be perceived as a blend of the two, does not appear as an intuitive blueprint of the brain. Moreover, it has been known for a while in network science that many different topological arrangements are possible within the confines of the small-world properties. For instance, small-world networks can show diversity with respect to how their topological features change across scales (Amaral et al. 2000) and possess or lack a modular structure (Meunier et al. 2010). Thus, the small-world structure can co-exist with, but does not necessarily entail, diverse topological properties and dynamic features.

What are characteristic topological features of brain networks? Ubiquitously observed features are a heterogeneous, non-random degree distribution, resulting in some nodes with more connections than others, so-called hubs (Sporns et al. 2007), as well as modules of connections, in which some nodes are more frequently linked with each other than with the rest of the network (Hilgetag et al. 2000; Sporns et al. 2004; Bullmore and Sporns 2009). These features may be combined, in so-called hub modules or rich clubs (Zamora-López et al. 2010; van den Heuvel and Sporns 2011), and repeated across several scales of organization, resulting in hierarchical network arrangements (Sporns 2006; Müller-Linow et al. 2008; Kaiser and Hilgetag 2010; Fig. 1b). Such topological aspects appear highly relevant for the organization of dynamic patterns observed in the networks (Hütt et al. 2014). In particular, activity patterns are strongly shaped by modular and hub features of cortical connections (Müller-Linow et al. 2008; Garcia et al. 2012; Gómez-Gardeñes et al. 2010).

Another global characterization of the organization of neural networks is provided by the so-called topological dimension which describes how quickly the whole network can be accessed from any of its nodes (Moretti and Muñoz 2013). This measure is not simply equivalent to path length, because the average path length does not specifically capture how quickly local node neighborhoods grow. Thus, networks with similar characteristic path length can have a different topological dimension, due to their distinct path length distributions. In classical small-world networks as described by Watts and Strogatz (1998; Fig. 1a), the networks are very well connected globally via the random network shortcuts. This means that the number of accessible nodes grows exponentially with the distance of steps from an initial node, formally corresponding to an infinite topological dimension (while ignoring finite-size effects, which mean that the dimension of finite networks is always finite). By contrast, the finite topological dimension that is the defining feature of so-called large-world networks (Moretti and Muñoz 2013) implies that some parts of the network are relatively inaccessible. The topological dimension, thus, offers an alternative perspective on the global organization of brain connectivity. Importantly, the characterization of large-world networks by their topological dimension and the traditional definition of small-world networks by features of clustering and short paths are not mutually exclusive. Nonetheless, they have different consequences for the global dynamics and ultimately the function of networks, as discussed further below. However, before entering this discussion, it is worth considering some practical aspects of gathering and analyzing brain network data that have implications for the inferred network organization.

## Small-worldness depends on the aperture of empirical studies

Brain connectivity is traditionally established by invasive histochemical tracing of connections in the brains of animal models. Extensive collations of such data for the macaque monkey cortex (Felleman and Van Essen 1991; Stephan et al. 2001) or for the cat (Scannell et al. 1999) formally show a small-world organization, although based on an arrangement of multiple, interlinked network modules, rather than a classical regular-with-random-links small-world network (Hilgetag and Kaiser 2004). More recently, however, extended quantitative compilations of connectivity data for the macaque cortex (Markov et al. 2014) appear to suggest a different picture. In particular, these data imply a very high density of cortico-cortical connections, with more than 60 % of the possible connections actually existing (Markov et al. 2013). Such high density renders the networks featureless, also in terms of a small-world organization, as the clustering and path lengths of these densely connected networks do not differ from those of same-size comparison networks (matched in the number of nodes, edges and degree distribution).

The measured network density is influenced by a number of factors, such as the amount of injected tracer, the thoroughness of sampling of labeled sections, or by treating projections as existing pathways no matter if they involve many or few fibers. The density of neural paths varies considerably, over five or more orders of magnitude, that is, from fewer than 10 to more than 100,000 labeled projection neurons (Markov et al. 2011). While it is still unclear how much the physiological or functional impact of a projection changes with its fiber density (e.g., Vanduffel et al. 1997), such graded networks might be more suitably analyzed by approaches that account for the differential weight of pathways (Rubinov and Sporns 2011). The issue is underlined by the fact that examples can be found where brain networks resemble a large-world network when only the stronger connections are taken into account, while incorporating the weakest connections shrinks them to a small-world network (Gallos et al. 2012). Thus, an analysis that takes into account the weight of the connections, especially in very dense networks, and employs weighted versions of network metrics including the small-world index (e.g. Bolaños et al. 2013) can be instructive.

Connectivity data for the human brain can be inferred from in vivo imaging techniques such as diffusion weighted imaging (DWI). However, these approaches face problems of limited specificity and sensitivity (Thomas et al. 2014). Hence, some connections might be unresolved by such methods (e.g., Zalesky and Fornito 2009; Li et al. 2012). Generally, experimental approaches for discovering networks have apertures that are tuned to particular scales and data features. Therefore, not all networks at all scales are accessible with the same method (such as electron microscopy or DWI). An additional methodological caveat is the application of thresholds to such in vivo data to create networks that can be analyzed (Rubinov and Sporns 2010), which discards weak existing connections. Such empirical limitations may lead to inaccurate brain network representations, including evidence of small-worldness.

Another important factor determining the density of links is the parcellation of the network nodes. The coarser the parcellation, the denser the network will appear, while a finer parcellation results in sparser connectivity. For instance, assembling the human connectome from diffusion data results in a density of 26 % at a coarse parcellation, whereas a high resolution parcellation results in a density of just 3 % (Samu et al. 2014). Moreover, if a relatively coarse parcellation scheme, such as the Regional Map (Kötter and Wanke 2005), is employed for representing connections from the CoCoMac primate connectivity database (http://cocomac.g-node.org), a very high network density is obtained (i.e. 79 %; Goulas et al. 2014). This density is much higher than the one obtained for using the same database with a different, more fine-grained parcellation scheme (i.e. ~1 %; Modha and Singh 2010) and very close to the high density of the primate cortico-cortical network estimated in more recent studies (Markov et al. 2014). The parcellation coarseness also leads to systematic changes in the small-worldness of the brain, with more finely grained networks showing a higher small-world index (Zalesky et al. 2010).

That begs the question, what is the neural network density and topology of the brain at the finest, cellular, level of parcellation? On average, the density of human brain connectivity at the cellular level is very sparse. The average number of synapses of neurons (~10^4^) (Braitenberg and Schüz 1998) divided by the number of neural elements (~10^10^) (Herculano-Houzel 2012) results in a very low average probability of any two neurons in the brain making contact (10^−6^), implying a highly dispersed network. The dispersion may be ameliorated via the local clustering of connectivity, for instance, in neural modules such as columns and layers so that within these compartments, the density is likely much higher (Markram 2006, but see Stepanyants et al. 2009). To be true, we do not really know the exact organization of brain networks at the cellular scale, since extensive empirical microconnectome data for the mammalian brain are still lacking. The one existing example of a complete neuronal microconnectome, of the nematode *C. elegans*, may be too small to be helpful here. While this network fits the small-world features of high clustering and short pathlengths (Watts and Strogatz 1998), it also appears to have a finite topological dimension (cf. Supplementary Figure). However, it is difficult to make a conclusive statement about this point, given the small number of just 302 neuronal network nodes that can be evaluated (White et al. 1986).

Nonetheless, it appears to be a reasonable guess that the organization of the mammalian brain follows a hierarchical organization (Meunier et al. 2009), with dense modules at the local level (cellular circuits, laminar compartments) that are encapsulated in increasingly larger modules (cortical columns, areas, whole lobes), but with very sparse overall connectivity (Hilgetag and Hütt 2014; Fig. 1b). This organization may produce different network topologies at different scales; for example, synaptic connectivity within local neuronal populations might form small-world or random networks. At the global scale, however, such a network may have a finite topological dimension (Moretti and Muñoz 2013), unlike classical small-world networks.

## Dynamics of small-world versus large-world networks

What are implications for brain dynamics, if the brain is organized as a small-world or a large-world network? As pointed out already by Watts and Strogatz (1998), the coexistence of high clustering and short average distances facilitates the integration and spreading of signals. Such a small-world organization might enhance dynamic complexity (Sporns et al. 2000), due to increased reentry (Edelman and Gally 2013) and signal integration. Additionally, network shortcuts can insert incoherent, remote information (‘topological noise’) into local coherent neighborhoods (Marr and Hütt 2006). Such shortcuts may enhance the robustness of classification or local decision-making tasks across networks (Moreira et al. 2004).

On the other hand, a non-modular small-world arrangement of the Watts and Strogatz type may not be the optimal topology for supporting limited self-sustained activity (Kaiser et al. 2007). Such sustained yet constrained network activity is an essential ingredient of healthy brain dynamics, by maintaining the balance between activity dissipating too quickly or becoming pathologically large. Sustained activity in an excitable system is also an important precondition for the phenomenon of criticality, the positioning of the system precisely at the boundary between order and chaos (or disorder). The feature of criticality has been associated with several desirable functional properties, such as a large dynamic range, high adaptability or optimal information processing (e.g., Shew and Plenz 2013). It has been observed that sustained network activity is better supported in hierarchical modular networks (Kaiser et al. 2007; Kaiser and Hilgetag 2010; Wang et al. 2011) than classical small-world networks. More generally, hierarchical modular networks that are large-world networks with a finite topological dimension possess so-called Griffith phases (Muñoz et al. 2010) that expand the parameter range of criticality. In contrast to the precise fine-tuning that is required in other systems to reach a critical point, for instance by carefully balancing local excitation and inhibition, an expanded range of criticality arises directly from the topology of large-world networks. This makes such systems dynamically appealing and robust, in addition to further aspects of structural robustness conferred by hierarchical modularity, such as the potential to assemble a large network from smaller subnetworks of similar organization, or split a large network into smaller units while maintaining their dynamical features (Robinson et al. 2009).

## Conclusions

To decide if the brain really adheres to a small-world organization and to understand the implications of this organization, one needs to take several theoretical and empirical aspects into account. The small-world property is influenced by practical aspects of analyzing brain connectivity; for example, whether connections are treated as weighted or as binary. Proper topological assessment requires the re-examination of weighted networks, an approach that entails a new definition of small-world properties (Bolaños et al. 2013). More generally, the small-world property depends on the aperture of experimental methods for studying brain connectivity, the coarseness of used parcellations and the resulting density of the studied brain networks. In that respect, recent compilations of brain connectivity at the macroscopic level do not appear to form small-world networks, due to their high density. If considered at the cellular level, brain networks are also unlikely to form classical small-world networks. While detailed empirical data are still lacking, a reasonable guess is that the large-scale neuronal networks of the brain are arranged as globally sparse hierarchical modular networks. Even if they fit the general features of local clustering and relatively short average paths, the small-world concept can miss the point of other essential topological properties of such brain networks, such as their finite topological dimension, which can also be used to characterize them as large-world networks.

This means that, at cellular resolution, the brain may be a large-world network, rather than a classical small-world network. Intriguingly, such a topology might fundamentally enhance the brain’s dynamic stability and information processing abilities. Thus, while most researchers have by now become accustomed to the small world of brain connectivity and might find it quite comforting, it may be just as exciting to step out and explore the large world of the brain.

## Electronic supplementary material

Below is the link to the electronic supplementary material.

Supplementary material 1 (PDF 32 kb)
