# Supplementary material for: Is the brain really a small-world network?
Source: Brain Struct Funct. 2015 Apr 18;221:2361–6. doi: 10.1007/s00429-015-1035-6 (PMC4853440; doi:10.1007/s00429-015-1035-6)
Supplement: Supplementary file 1 — Supplementary material 1 (PDF 32 kb) [file 429_2015_1035_MOESM1_ESM.pdf]

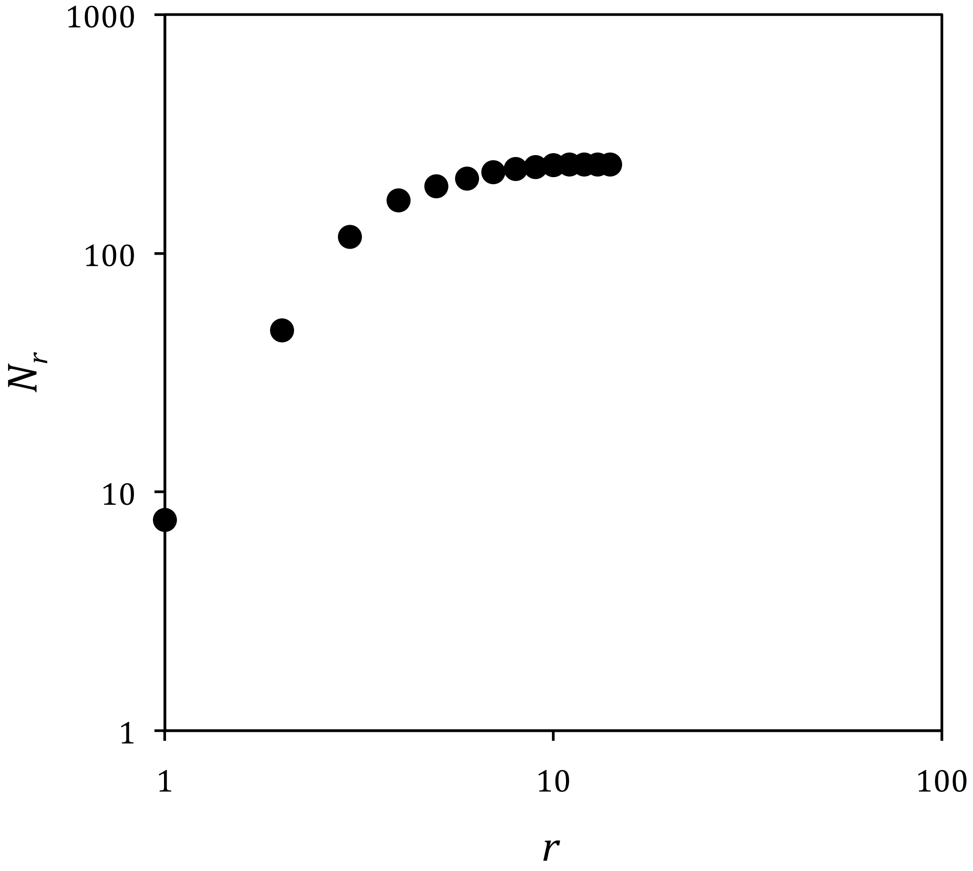

Hilgetag and Goulas (2015) Supplementary Figure. Topological dimension of the neuronal network of *C. elegans*. Average number of nodes N_r reached within r steps of starting from an initial node. The initial slope of the curve within a log-log plot suggest a finite topological dimension D, for $N_r \sim r^D$, rather than an infinite increase (cf. Moretti and Muñoz 2013). The asymptotic slope of the curve for larger r is due to the finite size of the network. Network data from http://www.dynamic-connectome.org.
